# Improving breaking bad news communication skills through stress arousal reappraisal and worked examples

**DOI:** 10.1111/medu.15658

**Published:** 2025-03-12

**Authors:** Michel Bosshard, Sissel Guttormsen, Urs Markus Nater, Felix Schmitz, Patrick Gomez, Christoph Berendonk

**Affiliations:** ^1^ Institute for Medical Education University of Bern Bern Switzerland; ^2^ Graduate School for Health Sciences University of Bern Bern Switzerland; ^3^ Department of Clinical and Health Psychology University of Vienna Vienna Austria; ^4^ University Research Platform “Stress of life (SOLE) – Processes and Mechanisms underlying everyday Life Stress” University of Vienna Vienna Austria; ^5^ Department of Occupational and Environmental Health Unisanté, Center for Primary Care and Public Health & University of Lausanne Lausanne Switzerland

## Abstract

**Introduction:**

Breaking bad news (BBN) is a distressing yet essential task in medicine, imposing emotional strain on both physicians and patients. Crucially, effective BBN relies on both verbal and nonverbal communication, which can be impaired by elevated stress associated with the task. Efficient teaching of communication skills continues to present a challenge, and the role of stress management in BBN encounters remains largely overlooked. In this study, we investigated the effects of stress arousal reappraisal (SAR; positive reframing of stress arousal) and worked example (WE; step‐by‐step demonstration of BBN) interventions on medical students' communication performance.

**Methods:**

This pre‐registered randomised controlled trial employed a 2 × 2 between‐subjects design to evaluate the individual and combined effects of SAR and WE interventions on the verbal and nonverbal communication performance of 221 third‐year medical students. To do so, students completed a 40‐min web‐based learning module before disclosing bad news to a simulated patient within a 12‐min consultation. Performances were videorecorded and assessed by three independent raters.

**Results:**

The WE intervention significantly improved both verbal and nonverbal communication performance, whereas the SAR intervention enhanced nonverbal communication only. Combining SAR with WE did not yield additional improvements in nonverbal communication beyond those achieved by either intervention alone.

**Discussion:**

These findings highlight the potential of both SAR and WE interventions to optimise resource‐intensive simulated BBN training. By demonstrating the efficacy of WE in improving both verbal and nonverbal communication, this study advances the literature on the application of WEs in the BBN context. Furthermore, this study is among the first to demonstrate the importance of stress coping in delivering bad news effectively. Given their low threshold, both SAR and WE interventions represent promising tools for equipping medical students with essential BBN communication skills and are well‐suited for integration into already time‐constrained medical curricula.

## Abstract 2

Breaking bad news is challenging; stress makes it harder. This research shows the value of stress arousal training and worked examples for helping to teach essential verbal and nonverbal communication skills.

## 1 INTRODUCTION

Breaking bad news (BBN) refers to the disclosure of serious diagnoses that results in negative, life‐altering circumstances for patients' present or future.^1^ Upon receiving bad news, patients often respond with intense emotions such as shock, isolation or grief.^2^ Effective BBN requires physicians to acknowledge and validate patient's emotions, convey the information with empathy without trivializing its severity and ensure that the patient fully understands the diagnosis and is informed about the next steps.^2^ Nonverbal communication, which refers to the transfer of information without the use of words but through nonverbal means^3^ (e.g. eye contact, posture, gestures and voice tone), plays an equally critical role in conveying empathy and attentiveness and establishing a trusting, fruitful doctor–patient relationship.^4^, ^5^ Effective communication of bad news is crucial for patients' satisfaction and long‐term health.^6^ While adhering to BBN principles can facilitate teaching of a foundational skillset, medical consultations profit from being viewed as dynamic, interactive processes in which physicians and patients collaboratively construct meaning. As both parties interpret and integrate verbal and nonverbal cues, a shared understanding is fostered that guides the conversation forward. Therefore, physicians should continuously assess the evolving context of the interaction and adapt their communicative behaviour accordingly.^7^

Due to its complexity and emotional demands, BBN is widely regarded as a distressing communication encounter for physicians.^8^ Effectively managing patients' emotions while ensuring the consultation proceeds appropriately represent main concerns, particularly for medical trainees.^9^ Medical students experience heightened stress during simulated BBN training.^10^ Even with BBN experience, this stress can divert the focus from BBN to stress regulation,^8^, ^11^, ^12^ leading physicians to miss patient cues and make disorganised, impulsive decisions, such as minimizing the severity of a diagnosis, providing misplaced optimism or resorting to abrupt and rushed (‘hit‐and‐run’) disclosures.^11^, ^13^ Despite limited experience, physicians may be expected to handle BBN responsibilities early in their career, emphasizing the need for dedicated BBN training programmes for medical trainees.^11^, ^14^

Teaching communication skills continues to present a challenge in medical education,^15^ and stress management programmes often face obstacles to implementation, such as time commitment.^16^ Importantly, although a large body of literature agrees on the stressful nature of BBN,^9^ educational efforts have mainly focused on teaching communication principles. There is a notable gap in research addressing strategies to support medical students and physicians in coping with stress, as well as the potential benefits of optimised stress management for BBN communication performance. Further, state‐of‐the‐art BBN training using simulated patients (SPs) is resource‐intensive.^17^ To maximise the value of these learning opportunities, developing low‐threshold tools that support both BBN skill‐building and stress management is essential.

The present study addresses these needs by adapting two well‐founded interventions—stress arousal reappraisal (SAR) and worked example (WE)‐based learning—to the BBN context.

SAR interventions are grounded in the biopsychosocial model of challenge and threat, which posits that evaluations of perceived task demands and personal coping resources determine whether individuals experience adaptive challenge or maladaptive threat states. Challenge states are present when perceived resources meet or exceed task demands, whereas threat states arise when perceived demands outweigh perceived resources.^18^ In this context, SAR involves reframing stress arousal itself as beneficial and functional, that is, as a resource for coping with task demands, thereby fostering a shift towards challenge‐type responses and consequently improved task performance,^19^, ^20^ including verbal and nonverbal competencies (e.g.^21^, ^22^). For instance, a fast heartbeat, often associated with anxiety, can instead be viewed as the body's way of increasing cardiac output and thus delivering additional oxygen to enable thriving in difficult situations. Importantly, SAR interventions have been shown to improve performance during social tasks requiring effective communication under stress.^23^, ^24^ However, to the best of our knowledge, SAR has not previously been applied in the context of medical communication (see previous works^25^ for a meta‐analysis).

Cognitive load theory suggests that effective learning depends on optimal allocation of limited cognitive resources.^26^ By minimizing extraneous load (i.e. unnecessary cognitive strain caused by poorly presented information), capacity can be freed for intrinsic load, which refers to the inherent complexity of the information. This reallocation enables deeper learning and understanding. WEs reduce task complexity by presenting clear step‐by‐step solutions, enabling mental schema acquisition.^27^ Schemas embody interrelated steps of a task and can be retrieved as a single unit from memory.^28^ This is especially valuable in stressful situations where cognitive resources are strained. Studies have demonstrated the efficacy of WE‐based learning in fostering BBN skills among medical students (e.g.^29^), although specific assessment of nonverbal communication is lacking.

Despite being based on different theoretical frameworks, SAR and WE may interact through shared mechanisms. For instance, similar to SAR, WE interventions may also enhance subjective resources by facilitating skill acquisition. In contrast, similar to WE interventions, SAR may also improve cognitive load by reducing anxiety related to psychosocial stress during BBN.^23^, ^30^ While the interventions address different aspects of BBN (i.e. stress and communication), it remains unclear whether their combined effects are synergistic or, conversely, nullify each other.

In this study, we evaluated the impact of SAR and WE interventions on verbal and nonverbal communication performance of medical students engaging in simulated BBN consultations for the first time. Based on the outlined theory, we tested following hypotheses: By reframing stress arousal as a resource, SAR improves verbal communication (1.1) and nonverbal communication (1.2). By optimizing cognitive load, WE‐based learning improves verbal communication (2.1) and nonverbal communication (2.2). Additionally, we explored the potential interaction effects between SAR and WE.

## 2 METHODS

The data for this study were obtained as part of a pre‐registered randomised controlled trial (ClinicalTrials.gov, NCT05037318). Details of the methodology are outlined in the study protocol.^31^

### 2.1 Study design

We employed a 2 × 2 between‐subjects design (SAR vs. No‐SAR, WE vs. No‐WE). Participants were stratified by sex and randomly assigned to one of four conditions (SAR‐only, WE‐only, SAR and WE, No‐intervention), using block randomisation with sizes of 4 or 8. While both participants and SPs were blinded to group assignment, experimenters were not, as they were responsible for setting up the condition‐specific modules.

### 2.2 Sample size calculation

Based on studies evaluating the effects of SAR in socio‐evaluative situations^25^ and WE in BBN context,^32^ we determined an effect size of *d* = 0.4^33^ to be appropriate and relevant. With a power of 0.8 and an alpha level of 0.05 (two‐tailed), 200 participants were necessary to test our hypotheses.

### 2.3 Participants

Two hundred twenty‐nine third‐year German speaking medical students were recruited via circular mail from four Swiss universities (Bern, *n* = 127; Basel, *n* = 44; Fribourg, *n* = 39; Zurich, *n* = 19). Students had not yet received formal training in BBN but had completed basic communication modules at their universities. The bad news scenario involved disclosing a trisomy 21 diagnosis. Although a prenatal setting may be challenging for third‐year students, we deliberately chose this scenario because it requires relatively little medical knowledge about the condition, especially in comparison to more commonly used examples such as cancer diagnosis. This allowed us to focus on the way the message was communicated rather than on students' medical knowledge. Furthermore, prenatal diagnosis is often overlooked in medical training, despite its increasing importance as diagnostics offer more options than ever. The mean age of participants was *M* = 22.42 (*SD* = 1.83) years with slightly over two‐thirds female participants (*n* = 158).

### 2.4 Study procedure

**FIGURE 1:**
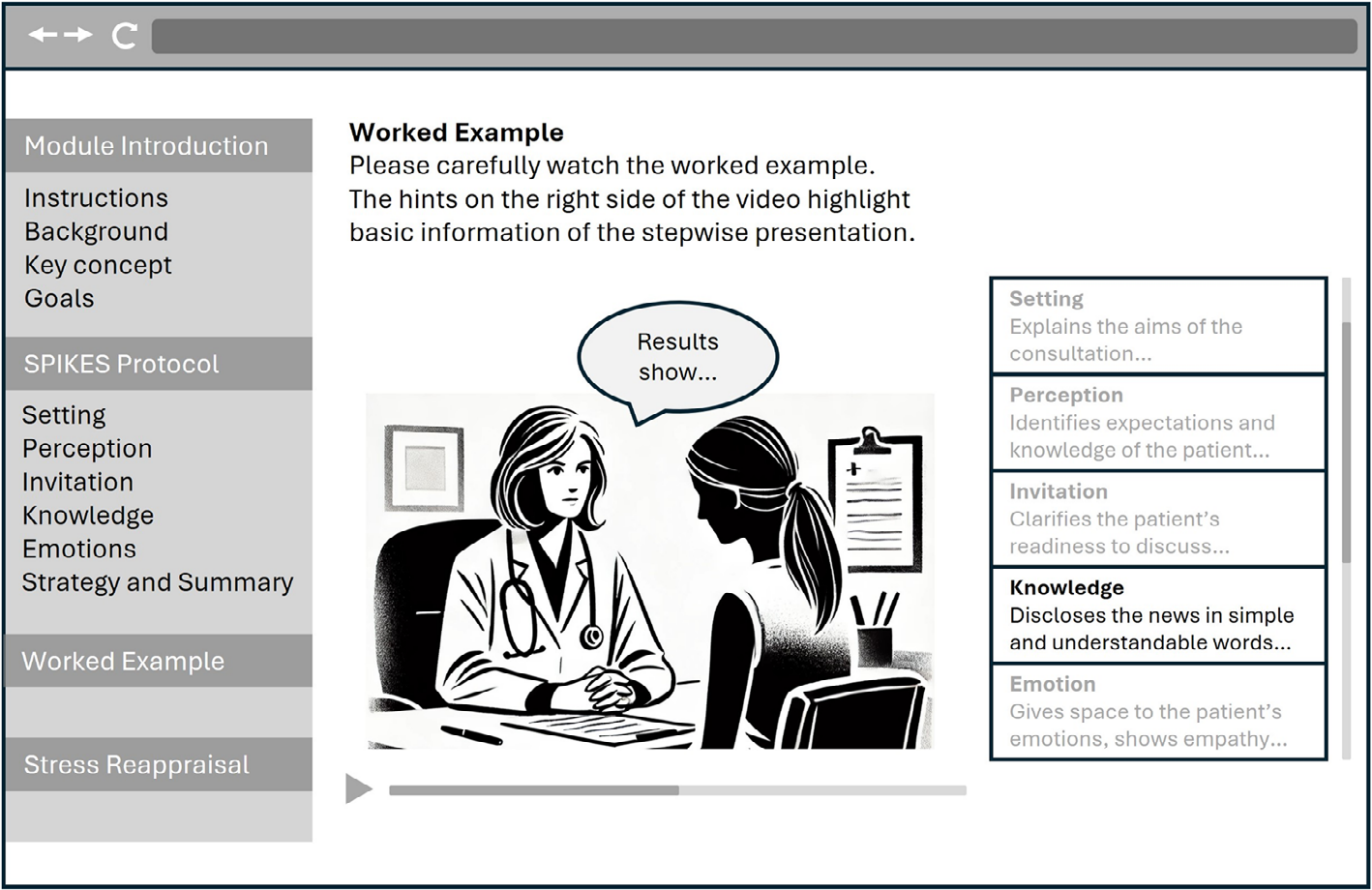
Illustration of the learning module. *Note.* The worked example and stress arousal reappraisal sections were only available for students in the respective groups. [Color figure can be viewed at wileyonlinelibrary.com]

**FIGURE 2:**
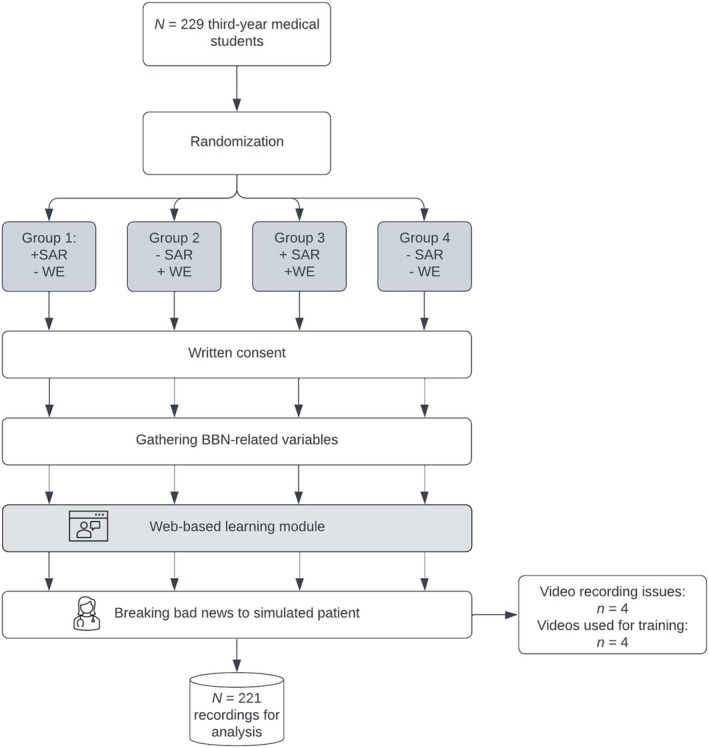
Participant flow chart. [Color figure can be viewed at wileyonlinelibrary.com]

For each participant, a 2‐h experimental session was scheduled at the Institute for Medical Education, University of Bern. At the beginning of the session, the experimenter explained the procedure, including that participants' BBN encounter would be videorecorded and their performance evaluated and obtained written informed consent. Participants were informed that the BBN consultation would take place in a prenatal setting. Following this, they completed questionnaires assessing their prior BBN experiences, perceived skills in BBN, interest in BBN and motivation to perform well on the task. Afterwards, all participants engaged in a 40‐min web‐based learning module (see Figure 1) that included a written BBN SPIKES protocol (Setting, Perception, Invitation, Knowledge, Emotions, Summary and Strategy^2^). This six‐step protocol covered gathering information, disclosing medical information, providing support and engaging the patient in planning the next steps. In addition, group‐specific interventions were incorporated into the learning module. After completing the module, participants received the specific task description that involved disclosing a Trisomy 21 diagnosis. They were given 5 min to prepare before conducting the BBN consultation with an SP. During the simulated BBN encounter, participants assumed the role of a junior doctor and were instructed to follow the SPIKES protocol presented in the learning module. The SP portrayed a pregnant woman and adhered to a detailed script, exhibiting emotional shock and detachment upon receiving the diagnosis. The participants had a maximum of 12 min to complete the task. On average, the encounter lasted 8 min 28 s (*SD* = 1 min 54 s). Students' performance was recorded using a video camera placed on a tripod next to the table, capturing both the student and the SP from a side angle, allowing for evaluation of full‐body nonverbal cues. To accommodate the large sample size, 11 SPs were employed to play the role of the pregnant woman, all of whom were trained by a professional SP instructor to ensure consistency across simulations. Figure 2 depicts the study procedure.

### 2.5 Measures

#### 2.5.1 BBN communication performance

Three independent raters (all female, between 23 and 25 years old) rated the communication performance of all participants using the video recordings. The raters were trained specifically for this task using the validated SPIKES scale,^32^ which was adapted to the prenatal setting (e.g. emphasizing the inclusion of both parents in the process), and an item assessing nonverbal performance (posture, gesture, eye contact, nodding, voice and rate of speech). The rating criteria are presented in Tables S1 and S2. Raters underwent two training sessions, during which they reviewed and assessed video recordings of participants' BBN encounters, which included one poor, two average and one good performance examples. An experimenter involved in rater training selected these examples. The assessments were thoroughly discussed to ensure consensus among raters. Each element of the SPIKES protocol (Setting, Perception, Invitation, Knowledge, Emotions, Strategy and Summary) and the nonverbal communication item were evaluated on a bipolar 5‐point scale (1 to 5), with higher values indicating better performance. For the analysis, verbal performance was calculated as the mean of the six SPIKES items, while nonverbal performance was calculated as a separate item for each rater.

#### 2.5.2 BBN‐related variables

We assessed practical and theoretical BBN experience, perceived skills and interest in BBN, and motivation to perform well on the BBN task. These items are described in detail in the Supporting Information.

### 2.6 Interventions

#### 2.6.1 SAR

The SAR intervention was based on previous research^23^, ^34^, ^35^ and consisted of a 7‐min screencast. The intervention explained the functionality of various bodily stress responses. For example, students were taught to view an increased heart rate during the BBN encounter as a positive sign that their body is providing more blood and oxygen to help them handle the task. The students were then asked to reflect on past and possible future scenarios, in which stress responses may enhance their performance. Finally, before participants engaged in the BBN task, they were encouraged to perceive their arousal as beneficial for their communication. The control screencast for the two groups not receiving the SAR intervention was a 7‐min video on neurocognitive learning principles (see previous works^34^ for a similar approach). Both screencasts are available in the OSF repository (https://osf.io/9aqwn/).

#### 2.6.2 WE‐based learning

The WE was presented as a 10‐min video, in which an expert physician demonstrated effective BBN to an SP. The physician followed the SPIKES protocol, providing examples for each step. Throughout the video, textual hints were displayed to inform about the content of each step (see Figure 1). The two groups not receiving the WE intervention were given an additional 10 min to work on the SPIKES protocol.

### 2.7 Statistical analysis

Due to technical difficulties, video recordings for four participants were unavailable. Additionally, the recordings of four participants were utilised during rater training. Thus, we excluded these eight participants from the statistical analysis, leaving a total of 221 observations.

The statistical models were computed with R^36^ (version 4.3.1) package *nlme* ^37^ (version 3.1.164) and *emmeans* ^38^ (version 1.10.0) for post hoc contrasts. The effects of SAR and WE on the verbal and nonverbal communication performance were analysed using multilevel mixed‐effects linear regressions based on restricted maximum likelihood. The models included fixed effects for the interventions (SAR/No‐SAR; WE/No‐WE) and their interaction (SAR × WE). Post hoc analysis of significant two‐way interactions focused on the effect of each intervention in the presence and absence of the other intervention. In preliminary analyses, we assessed the effect of the five BBN‐related variables on task performance (see Table S3). Only motivation to perform well significantly predicted nonverbal communication performance and was thus included in the corresponding model as a control variable.

Crossed random intercepts were specified for participants and raters. Moreover, heterogeneous residual structure for raters was specified as this further improved model fit. These model specifications allow both between‐participants and between‐raters variances to be appropriately accounted for.^39^ Model assumptions regarding linearity, normality and homoscedasticity were visually confirmed with residual versus fitted values plots, QQ‐plots and random effect plots.

## 3 RESULTS

The results regarding sociodemographic and BBN‐related variables for each group are presented in Table S4.

### 3.1 Verbal communication performance

**FIGURE 3:**
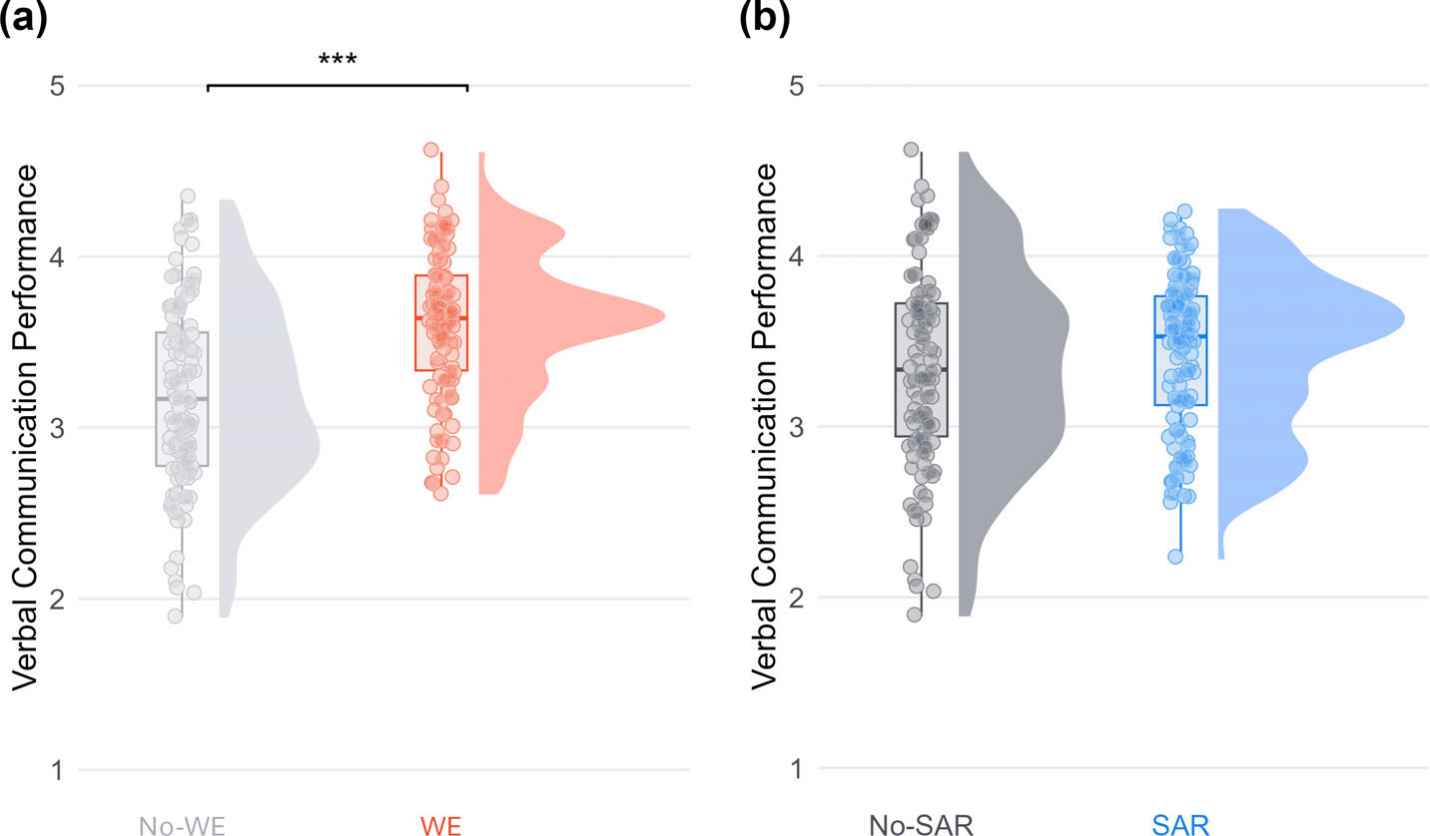
Rain cloud plot of the verbal communication performance for the main effects. *Note.* Distribution of raw data for the worked example (WE) groups and No‐WE groups (a), and for the stress arousal reappraisal (SAR) groups and No‐SAR groups (b). The boxplot indicates data distribution around the median with whiskers representing 1.5× the interquartile range. *** *p* < 0.001. [Color figure can be viewed at wileyonlinelibrary.com]

The WE groups (WE‐only and SAR and WE; *M* = 3.56, *SE* = 0.04) performed significantly better on the verbal aspects of the BBN task than the No‐WE groups (SAR‐only and No‐intervention; *M* = 3.20, *SE* = 0.04; *B* = 0.37, *SE* = 0.05, *p* < 0.001; see Figure 3a). There was no significant difference between the SAR groups (SAR‐only and SAR and WE; *M* = 3.40, *SE* = 0.04) and the No‐SAR groups (WE‐only and No‐intervention; *M* = 3.36, *SE* = 0.04; *B* = 0.04, *SE* = 0.05, *p* = 0.38; see Figure 3b). The SAR × WE interaction was not significant (*B* = −0.13, *SE* = 0.10, *p* = 0.20). The full model is reported in Table S5.

### 3.2 Nonverbal communication performance

**FIGURE 4:**
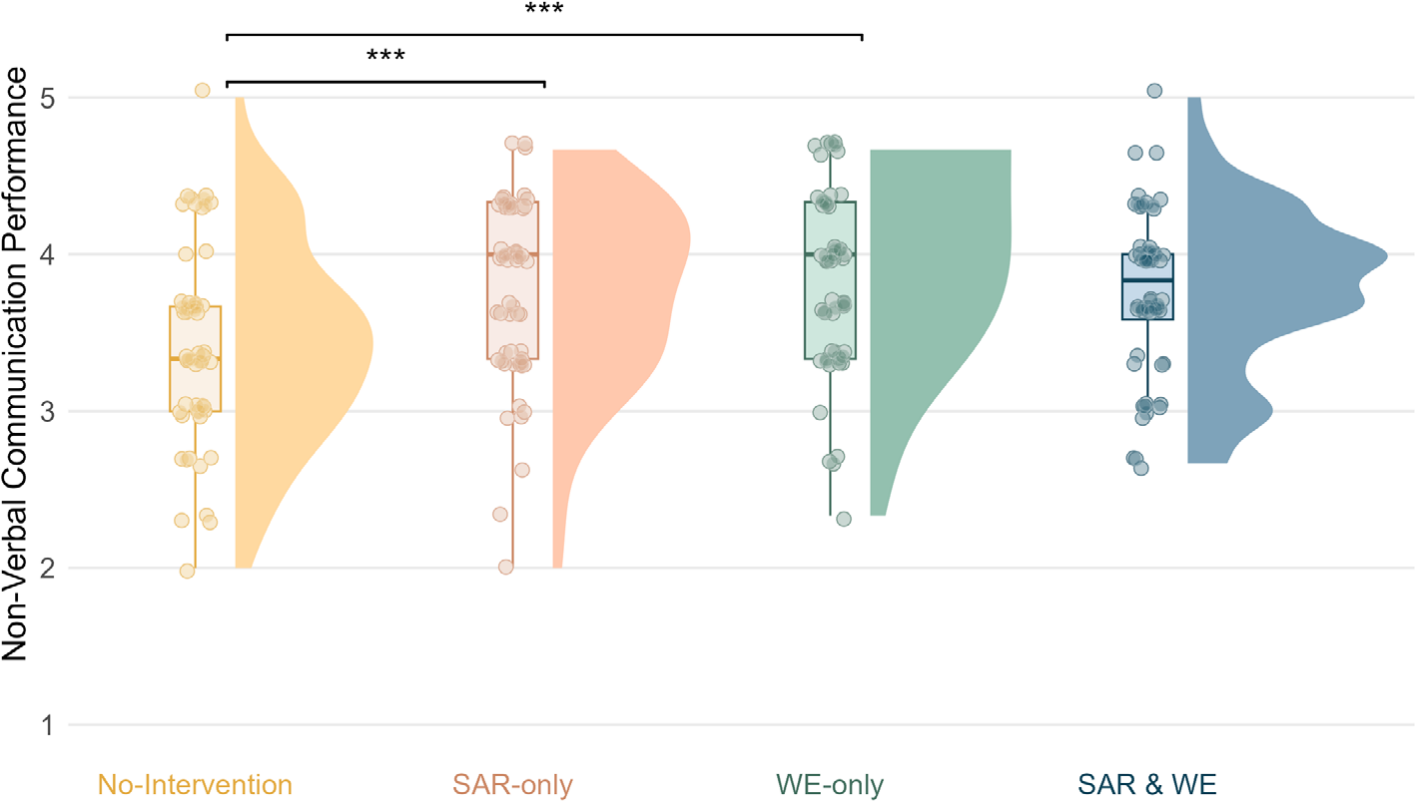
Rain cloud plot of the nonverbal communication performance for each group in the post hoc analysis. *Note.* The boxplot indicates the distribution of the raw data around the median with whiskers representing 1.5× the interquartile range. *** *p* < 0.001. [Color figure can be viewed at wileyonlinelibrary.com]

The WE groups (*M* = 3.90, *SE* = 0.04) performed significantly better in nonverbal communication than the No‐WE groups (*M* = 3.71, *SE* = 0.04; *B* = 0.19, *SE* = 0.06, *p* = 0.001). Similarly, the SAR groups (*M* = 3.87, *SE* = 0.04) performed significantly better than the No‐SAR groups (*M* = 3.75, *SE* = 0.04; *B* = 0.12, *SE* = 0.06, *p* = 0.039). These main effects were qualified by a significant SAR × WE interaction (*B* = −0.32, *SE* = 0.11, *p* = 0.005). Post hoc analysis of the SAR × WE interaction revealed that the WE effect was significant for the comparison between the WE‐only group (*M* = 3.93, *SE* = 0.06) and the No‐intervention group (*M* = 3.57, *SE* = 0.06; *B* = 0.35, *SE* = 0.08, *p* < 0.001), but not between the SAR and WE group (*M* = 3.88, *SE* = 0.06) and the SAR‐only group (*M* = 3.85, *SE* = 0.06; *B* = 0.03, *SE* = 0.08, *p* = 0.70). Similarly, the SAR effect was significant for the comparison between the SAR‐only group and the No‐intervention group (*B* = 0.28, *SE* = 0.08, *p* < 0.001), but not between the SAR and WE group and WE‐only group (*B* = −0.04, *SE* = 0.08, *p* = 0.62). The full model is reported in Table S5. Figure 4 illustrates the distribution of the nonverbal communication for each of these groups.

## 4 DISCUSSION

This study examined the effects of SAR instructions and WE‐based learning on the verbal and nonverbal communication performance of medical students in simulated BBN scenarios. The findings indicate that only the WE intervention enhanced verbal communication performance, whereas both SAR and WE independently improved nonverbal communication. Combining the two interventions did not yield additional benefits.

### 4.1 Effects of the SAR intervention on communication performance

Contrary to hypothesis 1.1, the SAR intervention had no significant effect on verbal aspects of the consultation. However, consistent with hypothesis 1.2, it did improve nonverbal communication. This result provides further insights into SAR's varying effects across different communication tasks (e.g.^24^, ^40^, ^41^). We propose two explanations for the differential effects of SAR on verbal and nonverbal communication. First, stress tends to manifest more clearly in nonverbal behaviours, such as avoiding eye contact or fidgeting, which are harder to consciously regulate than verbal communication.^42^ Consequently, as a stress coping strategy, SAR may particularly benefit nonverbal communication. Second, SAR's focus on bodily arousal might have heightened participants' awareness of their nonverbal behaviours, prompting greater efforts to regulate them more effectively.

Notably, combining SAR with WE did not yield additional benefits compared to WE alone. This may reflect the constraints of cognitive capacity, as participants were required to process and internalise two interventions alongside the SPIKES protocol within a 40‐min session. Extending the training duration or delivering the interventions in separate sessions might alleviate this issue, allowing learners to process and integrate both interventions more effectively.

### 4.2 Effects of WE‐based learning on communication performance

As hypothesised, the WE intervention significantly enhanced both verbal (hypothesis 2.1) and nonverbal communication (hypothesis 2.2). As with SAR, the combined SAR and WE intervention did not provide additional nonverbal communication benefits over SAR alone.

Within the BBN context, the positive effect of WE on nonverbal communication expands upon previous research, which has predominantly focused on its benefits on verbal communication.^29^, ^32^ This effect may stem from participants mimicking the expert's nonverbal behaviour demonstrated in the WE video, even without explicit instructions. Alternatively, the improved verbal communication skills among WE participants may have bolstered their confidence, leading to more assured body language.

### 4.3 Strengths, limitations and outlook

This study has several notable strengths: It was conducted as a pre‐registered randomised controlled trial, ensuring methodological rigour and transparency. It investigated the individual and combined effects of the SAR and WE interventions on verbal and nonverbal communication using validated assessment tools, with a large sample of participants inexperienced in BBN, ensuring a robust evaluation. Additionally, the use of two interventions grounded in distinct theoretical frameworks allowed the study to address the challenges of BBN through complementary pathways: SAR targeted stress management during BBN, while WE facilitated skill acquisition by fostering cognitive schema development.

Several limitations should be considered when interpreting our findings. First, the study was conducted in a simulated environment, which may limit the generalizability of the results to real‐world settings. Nonetheless, evidence suggests that simulation‐based training translates well to clinical performance.^43^ Moreover, simulations provide a controlled, safe space for students to practise and develop communication and stress management skills without jeopardizing patient well‐being. Second, the study focused exclusively on novice learners. While this aligns with the WE intervention, which is tailored to beginners,^26^ and SAR's reliance on participants experiencing stress,^20^ our findings may not readily apply to more experienced professionals.

Future research could explore the applicability of these interventions to more experienced populations, investigate their long‐term effectiveness and explore how the found effects translate to patient satisfaction and health. Furthermore, it would be valuable to investigate whether SAR has broader applications, including spillover effects on other high‐stress medical tasks, since its principles are not exclusive to BBN.

### 4.4 Implications and conclusion

This study demonstrated that both SAR and WE‐based learning are effective, time‐efficient strategies for supporting novice students in learning BBN. Based on our findings, the WE‐only intervention appears to be the most efficient strategy, as it demonstrated benefits for both verbal and nonverbal communication, without additional gains from SAR. However, we would not exclude the possibility that the combination of SAR and WE may still offer additive benefits for nonverbal communication, if training sessions are extended or the interventions are delivered separately.

Moreover, SAR's positive impact on nonverbal communication highlights the importance of stress management in BBN—an area that has historically been overlooked. SAR, being a simple, cost‐effective and easily deployable intervention, holds promise not only for BBN but also for addressing other stressful tasks in medical education and practice. In conclusion, SAR and WE leverage distinct mechanisms to support communication performance under stress, making them potentially valuable tools for inclusion in dense medical curricula.

## AUTHOR CONTRIBUTIONS

**Michel Bosshard**: Methodology; writing—original draft; visualization; formal analysis; investigation; resources. **Sissel Guttormsen**: Conceptualization; methodology; writing—review and editing; funding acquisition. **Urs Nater**: Conceptualization; methodology; writing—review and editing; funding acquisition. **Felix Schmitz**: Conceptualization; methodology; writing—review and editing; supervision; project administration; funding acquisition; formal analysis. **Patrick Gomez**: Conceptualization; methodology; formal analysis; writing—review and editing; supervision; funding acquisition. **Christoph Berendonk**: Conceptualization; methodology; writing—review and editing; supervision; project administration; funding acquisition. All authors have approved the final version of the manuscript to be published and agreed to be accountable for all aspects of the work in ensuring that questions related to the accuracy or integrity of any part of the work are appropriately investigated and resolved.

## CONFLICT OF INTEREST STATEMENT

The authors have no competing interests to declare.

## ETHICAL APPROVAL

The study was approved by the cantonal ethics committee of Bern (2021‐02098). The study was conducted according to the Declaration of Helsinki, the ICH‐Good Clinical Practice Guidelines, and the Swiss Federal Human Research Act. Informed consent was obtained from each participant before they were submitted to any study procedure. Participants received a compensation of 150 Swiss francs upon completion of the experiment.

## Supporting information


**Table S1.** SPIKES prenatal diagnostic rater‐scale and non‐verbal communication (English translation).
**Table S2.** SPIKES prenatal diagnostic rater‐scale and non‐verbal communication (German original).
**Table S3.** Preliminary analysis of potential control variables.
**Table S4.** Sociodemographic and BBN‐related variables for the four experimental groups.
**Table S5.** Multilevel mixed‐effects linear regressions for verbal communication performance and non‐verbal communication performance.

## Data Availability

The datasets generated and analysed during the current study are available in the OSF repository (https://osf.io/9aqwn/).
