# Supplementary material for: Improving breaking bad news communication skills through stress arousal reappraisal and worked examples
Source: Med Educ. 2025 Mar 12;59(8):853–61. doi: 10.1111/medu.15658 (PMC12242891; doi:10.1111/medu.15658)
Supplement: Supplementary file 1 — Table S1. SPIKES prenatal diagnostic rater‐scale and non‐verbal communication (English translation). Table S2. SPIKES prenatal diagnostic rater‐scale and non‐verbal communication (German original). Table S3. Preliminary analysis of potential control variables. Table S4. Sociodemographic and BBN‐related variables for the four experimental groups. Table S5. Multilevel mixed‐effects linear regressions for verbal communication performance and non‐verbal communication performance. [file MEDU-59-853-s001.docx]

**Improving breaking bad news communication skills through stress arousal reappraisal and worked examples**

**Supporting Information**

Michel Bosshard^1,2*^, Sissel Guttormsen^1^, Urs Markus Nater^3,4^, Felix Schmitz^1^ ^†^, Patrick Gomez^5^ ^†^, Christoph Berendonk^1^ ^†^

^1^Institute for Medical Education, University of Bern, Bern, Switzerland

^2^Graduate School for Health Sciences, University of Bern, Bern, Switzerland

^3^Department of Clinical and Health Psychology, University of Vienna, Vienna, Austria

^4^University Research Platform “Stress of life (SOLE) – Processes and Mechanisms underlying everyday Life Stress”, University of Vienna, Vienna, Austria

^5^ Department of Occupational and Environmental Health, Unisanté, Center for Primary Care and Public Health & University of Lausanne, Lausanne, Switzerland

† Felix Schmitz, Patrick Gomez and Christoph Berendonk share last authorship.

* Corresponding Author

**Prior Experience, Skills, Interest, and Motivation in BBN**

We measured previous practical and theoretical experience in BBN with one *yes/no* item each (“I have already gained practical experience in the field of ‘breaking bad news’ before taking part in today's study”, “I have already consulted learning material in the field of ‘breaking bad news’ before taking part in today’s study”). Three7-point scales were used to assess perceived BBN skills (“How high do you rate your ability to break bad news to patients?”; 1 *very low*, 7 *very high*), interest in BBN (“My interest in the topic of ‘breaking bad news’ is...”; 1 *very little interest*, 7 *very interested*), and motivation to perform well on the BBN task (“I am motivated to perform well in ‘breaking bad news’ as part of this study”; 1 *not at all motivated*, 7 *absolutely motivated*).

**Supplementary Table S1.** SPIKES prenatal diagnostic rater-scale and non-verbal communication (English translation)

| **SETTING**–the student… | | | | | | |
| --- | --- | --- | --- | --- | --- | --- |
| … does not introduce him/herself; does not offer a seat to the patient; does not seek eye contact; does not ask about the patient's current condition; does not inquire about a close/related person (partner); does not indicate that he/she is familiar with the case; does not clarify the goals of the conversation. | 1 | 2 | 3 | 4 | 5 | ...introduces him/herself by name and representative function; offers the patient a seat; seeks and maintains eye contact; asks about the patient's current condition; inquires about a close/ related person (partner); indicates that he/she is familiar with the case; clarifies the goals of the conversation. |
| **PERCEPTION**–the student… |  |  |  |  |  |  |
| Overall rating Perception | 1 | 2 | 3 | 4 | 5 |  |
| Phase 1 (before disclosing the diagnosis): ...does not inquire about the patient's understanding and expectations regarding the performed examinations (in particular implications of a widened nuchal fold and motivation for prenatal diagnostics). |  |  |  |  |  | Phase 1 (before disclosing the diagnosis): ...inquires about the patient's understanding and expectations regarding the performed examinations (in particular implications of a widened nuchal fold and motivation for prenatal diagnostics). |
| Phase 2 (after disclosing the diagnosis): ...does not assess the patient's attitude towards a child with Down syndrome. |  |  |  |  |  | Phase 2 (after disclosing the diagnosis):  ...assesses the patient's attitude towards a child with Down syndrome. |
| **INVITATION**–the student… |  |  |  |  |  |  |
| Overall rating Invitation | 1 | 2 | 3 | 4 | 5 |  |
| Phase 1 (before disclosing the diagnosis): ...communicates the diagnosis without first clarifying if the patient is ready to receive the diagnosis. |  |  |  |  |  | Phase 1 (before disclosing the diagnosis): ...clarifies if the patient is ready to receive the diagnosis, before communicating the diagnosis. |
| Phase 2 (after disclosing the diagnosis): ...does not clarify to what degree the patient wants to be informed about the diagnosis. |  |  |  |  |  | Phase 2 (after disclosing the diagnosis): ...clarifies to what degree the patient wants to be informed about the diagnosis. |

**Supplementary Table S1 (continued).**

| **KNOWLEDGE**–the student… |  |  |  |  |  |  |
| --- | --- | --- | --- | --- | --- | --- |
| ...delivers the bad news without announcing that he/she has bad news first; communicates the message in expert terminology, in one piece without pauses, does not give the patient an opportunity to ask questions; trivializes the diagnosis or is unnecessarily harsh; does not reassure that the patient has understood the message. | 1 | 2 | 3 | 4 | 5 | …first announces that he/she has bad news before delivering the bad news; communicates the diagnosis in clear, comprehensible language in small portions with pauses; provides the patient with an opportunity to ask questions; does not trivialize the diagnosis and is also not unnecessarily harsh; reassures that the patient has understood the message. |
| **EMOTIONS**–the student… |  |  |  |  |  |  |
| ...continues speaking directly after delivering the diagnosis, does not give room to the patient's emotions, does not listen; does not identify the patient's emotions or inquire about the patient's emotional state; does not legitimize the patient's emotions; does not show empathy. | 1 | 2 | 3 | 4 | 5 | ...is quiet after delivering the diagnosis, gives room to the patient's emotions, listens; identifies the patient's emotions or inquires about the patient's emotional state; legitimizes the patient's emotions; shows empathy |
| **STRATEGY and SUMMARY**–the student… | | | | | | |
| ...advises/urges that the patient makes a decision; does not communicate that he/she will continue to be available to the patient; does not arrange follow-up appointments/consultations, does not provide assistance (e.g., helps the patient to get home safe...); does not summarize key points. | 1 | 2 | 3 | 4 | 5 | ...decelerates the decision-making process (e.g. advises to process the diagnosis first, to inform and involve the partner, second consultation); informs the patient that he/she will continue to be available; arranges follow-up appointments/ consultations, provides support (e.g. helps the patient to get home safe...); summarizes essential points again. |
| **NON-VERBAL**– the student has displayed the following signals throughout the situation… | | | | | | |
| ... hunched posture & does not face the patient, unsteady posture (e.g., fidgeting), does not display non-verbal approval (e.g., nodding), no eye contact, inappropriate voice (e.g., mumbling), and speed of speech (too fast). | 1 | 2 | 3 | 4 | 5 | ...upright posture & faces patient, calm posture (e.g., not fidgeting), displays non-verbal approval (e.g., nodding), stable eye contact, appropriate voice and rate of speech. |

**Supplementary Table S2.** SPIKES prenatal diagnostic rater-scale and non-verbal communication (German original)

| **SETTING**–der/die Studierende… | | | | | | |
| --- | --- | --- | --- | --- | --- | --- |
| …stellt sich nicht vor; bietet der Patientin keinen Stuhl an; sucht keinen Augenkontakt; fragt nicht nach dem momentanen Befinden der Patientin; erkundigt sich nicht nach Bezugsperson (Partner); gibt nicht zu verstehen, dass er/sie mit dem Fall vertraut ist; klärt die Ziele der Konversation nicht. | 1 | 2 | 3 | 4 | 5 | …stellt sich mit Namen und Stellvertreter-Funktion vor; bietet der Patientin einen Stuhl an; sucht und hält Augenkontakt; fragt nach dem momentanen Befinden der Patientin; erkundigt sich nach Bezugsperson (Partner); gibt zu verstehen, dass er/sie mit dem Fall vertraut ist; klärt die Ziele der Konversation. |
| **PERCEPTION**–der/die Studierende… |  |  |  |  |  |  |
| Gesamtbewertung Perception | 1 | 2 | 3 | 4 | 5 |  |
| Phase 1 (vor Diagnose): …erfragt den Wissensstand und die Erwartungen der Patientin in Bezug auf die gelaufenen Abklärungen nicht (insbesondere Einordnung der verbreiterten Nackenfalte und Beweggründe für Pränataldiagnostik). |  |  |  |  |  | Phase 1 (vor Diagnose): …erfragt den Wissensstand und die Erwartungen der Patientin in Bezug auf die gelaufenen Abklärungen (insbesondere Einordnung der verbreiterten Nackenfalte und Beweggründe für Pränataldiagnostik). |
| Phase 2 (nach Eröffnung der Diagnose): …ermittelt die Einstellung der Patientin gegenüber einem Kind mit Downsyndrom nicht. |  |  |  |  |  | Phase 2 (nach Eröffnung der Diagnose):  …ermittelt die Einstellung der Patientin gegenüber einem Kind mit Downsyndrom. |
| **INVITATION**–der/die Studierende… |  |  |  |  |  |  |
| Gesamtbewertung Invitation | 1 | 2 | 3 | 4 | 5 |  |
| Phase 1 (vor Diagnose): …teilt die Diagnose mit, ohne vorher die Bereitschaft der Patientin zu klären. |  |  |  |  |  | Phase 1 (vor Diagnose): …klärt die Bereitschaft der Patientin die Diagnose jetzt (und ohne Partner) zu besprechen. |
| Phase 2 (nach Eröffnung der Diagnose): …klärt nicht ab, wieviel die Patientin über die Diagnose wissen will. |  |  |  |  |  | Phase 2 (nach Eröffnung der Diagnose): …klärt ab, wieviel die Patientin über die Diagnose wissen will. |

**Supplementary Table S2 (continued).**

| **KNOWLEDGE**–der/die Studierende… |  |  |  |  |  |  |
| --- | --- | --- | --- | --- | --- | --- |
| …überbringt die schlechte Nachricht, ohne diese mit einem Warnschuss anzukünden; übermittelt die Botschaft in Fachsprache, an einem Stück ohne Pausen, gibt der Patientin keine Gelegenheit Fragen zu stellen; beschönigt die Diagnose oder ist unnötig harsch; vergewissert sich nicht, ob die Patientin die Nachricht verstanden hat. | 1 | 2 | 3 | 4 | 5 | …kündigt die schlechte Nachricht mit einem Warnschuss an; übermittelt die Botschaft dann in klarer, verständlicher Sprache in kleinen Portionen mit Pausen dazwischen, gibt der Patientin Gelegenheit Fragen zu stellen; beschönigt die Diagnose nicht, ist aber auch nicht unnötig harsch; vergewissert sich, dass die Patientin die Nachricht verstanden hat. |
| **EMOTIONS**–der/die Studierende… |  |  |  |  |  |  |
| …spricht nach der Botschaft direkt weiter, gibt den Emotionen der Patientin keinen Raum, hört nicht zu; benennt die Emotionen der Patientin nicht oder fragt nicht nach dem emotionalen Zustand der Patientin; legitimiert die Emotionen der Patientin nicht; zeigt keine Empathie. | 1 | 2 | 3 | 4 | 5 | …schweigt nach der Botschaft, gibt den Emotionen der Patientin Raum, hört zu; benennt die Emotionen der Patientin oder fragt nach dem emotionalen Zustand der Patientin; legitimiert die Emotionen der Patientin; zeigt Empathie |
| **STRATEGY and SUMMARY**–der/die Studierende… | | | | | | |
| …rät/drängt zu einer Entscheidungstreffung; teilt nicht mit, dass er/sie der Patientin weiter zur Verfügung steht; arrangiert keine Folgetermine/Sprechstunden, sorgt nicht für Unterstützung (z.B. nach Hause kommen..); fasst die wesentlichen Punkte nicht zusammen. | 1 | 2 | 3 | 4 | 5 | …entschleunigt die Entscheidungstreffung (z.B. rät dazu, die Nachricht erst zu verarbeiten, den Partner zu informieren und einzubinden, Zweitgespräch); teilt mit, dass er/sie der Patientin weiter zur Verfügung steht; arrangiert Folgetermine/Sprechstunden, sorgt für Unterstützung (z.B. nach Hause kommen..); fasst wesentliche Punkte nochmals zusammen. |
| **NON-VERBAL**–der/die Studierende hat während der gesamten Situation folgende Signale gezeigt… | | | | | | |
| …gekrümmte & abgewandte Körperhaltung, unruhige Grundhaltung (z.B. Zappeln), benutzt keine Zeichen der non-verbalen Zustimmung (z.B. Nicken), kein Augenkontakt, unangebrachte Stimmlage (z.B. Nuscheln) und Sprechtempo (zu schnell). | 1 | 2 | 3 | 4 | 5 | …aufrechte & zugewandte Körperhaltung, gelassene Grundhaltung (z.B. nicht Zappeln), benutzt Zeichen der non-verbalen Zustimmung (z.B. Nicken), stabiler Augenkontakt, angebrachte Stimmlage und Sprechtempo. |

**Supplementary Table S3.** Preliminary analysis of potential control variables.

| Outcome | Predictor | *F*(1, 219) | *p* |
| --- | --- | --- | --- |
| Verbal Communication | Perceived BBN skills | 0.35 | .56 |
|  | BBN theoretical experience | 0.85 | .36 |
|  | BBN practical experience | 1.50 | .22 |
|  | BBN interest | 1.00 | .32 |
|  | BBN motivation | 0.01 | .97 |
| Non-Verbal Communication | Perceived BBN skills | 2.08 | .15 |
|  | BBN theoretical experience | 0.17 | .68 |
|  | BBN practical experience | 0.14 | .71 |
|  | BBN interest | 0.13 | .72 |
|  | BBN motivation | **4.77** | **.03** |

*Note.* Effects are based on multilevel mixed-effects linear regression models only including the respective variable as fixed effect. Significant effects are highlighted in bold.

**Supplementary Table S4.** Sociodemographic and BBN-related variables for the four experimental groups.

|  | SAR-only  (*n* = 54) | WE-only  (*n* = 56) | SAR & WE (*n* = 56) | No-intervention  (*n* = 55) | Effect |
| --- | --- | --- | --- | --- | --- |
| Sex  (% female) | 70.4 | 67.9 | 67.9 | 67.3 | *X*^2^ (3, *N* = 221) = 0.14, *p* = .99 |
| Age (in years) | 22.44 (2.18) | 22.21 (1.33) | 22.63 (1.81) | 22.42 (1.97) | *F*(3, 217) = 0.47, *p* = .71 |
| Perceived BBN skills | 4.1 (0.8) | 4.1 (1.0) | 3.9 (0.9) | 3.9 (0.9) | *F*(3, 217) = 0.66, *p* = .58 |
| BBN theoretical experience  (% yes) | 16.7 | 8.9 | 7.1 | 9.1 | *X*^2^ (3, *N* = 221) = 3.14, *p* = .37 |
| BBN practical experience  (% yes) | 18.6 | 7.1 | 8.9 | 9.1 | *X*^2^ (3, *N* = 221) = 4.46, *p* = .22 |
| BBN interest | 5.9 (0.9) | 5.9 (0.9) | 5.9 (0.8) | 5.6 (0.8) | *F*(3, 217) = 1.46, *p* = .23 |
| BBN motivation | 6.6 (0.6) | 6.1 (0.8) | 6.4 (0.7) | 6.3 (0.9) | *F*(3, 217) = 3.83, *p* = .010 |
| BBN task duration  (in seconds) | 528 (113) | 511 (103) | 506 (111) | 492 (118) | *F*(3, 217) = 0.97, *p* = .41 |

*Note.* Mean values with standard deviation in parentheses.

**Supplementary Table S5.** Multilevel mixed-effects linear regressions for verbal communication performance and non-verbal communication performance.

| Fixed Effect | Verbal Performance | | | Non-verbal Performance | | |
| --- | --- | --- | --- | --- | --- | --- |
|  | Beta | *SE* | *p* | Beta | *SE* | *p* |
| Intercept | 3.38 | 0.02 | < .001 | 3.23 | 0.24 | < .001 |
| SAR ^a^ | 0.04 | 0.05 | .38 | **0.12** | **0.06** | **.039** |
| WE ^a^ | **0.37** | **0.05** | **< .001** | **0.19** | **0.06** | **.001** |
| SAR × WE | -0.13 | 0.10 | .20 | **-0.32** | **0.11** | **.005** |
| BBN Motivation ^b^ |  |  |  | **0.07** | **0.03** | **.018** |

*Note.* BBN Motivation was included in the non-verbal communication performance model because it significantly affected the outcome (see Table S3). ^a^ Both interventions were effect-coded (SAR = 0.5, No-SAR = -0.5; WE = 0.5, No-WE = -0.5) to ensure that the main effects remained interpretable even in the presence of the SAR × WE interaction. ^b^ BBN Motivation is grand mean centered. Significant effects are highlighted in bold.
